# Commentary: *In Utero* Exposure to Metformin Reduces the Fertility of Male Offspring in Adulthood

**DOI:** 10.3389/fendo.2021.815532

**Published:** 2022-01-03

**Authors:** Deborah Cavalcanti, Emmanuel Bujold

**Affiliations:** ^1^ Research Center of the Centre Hospitalier Universitaire (CHU) de Quebec – Université Laval, Quebec City, QC, Canada; ^2^ Department of Obstetrics and Gynecology, Faculty of Medicine, Université Laval, Quebec City, QC, Canada

**Keywords:** pregnancy, metformin, fetus, preeclampsia, diabetes

It was with great interest that we read the manuscript of Faure et al. evaluating the impact of metformin during mice pregnancies (1). We are concerned that the interpretation of the results yielded by this study may confuse readers due to the suggestion of a deleterious effect in the human male fetus. Indeed, a growing body of clinical scientific evidence suggests that the use of metformin in obese pregnant women may reduce the risk of placenta-mediated complications such as preeclampsia (2). In this context, it becomes essential for the scientific community, and for clinician-researchers involved in maternal health to rely on the most truthful and accurate information possible to adequately inform pregnant women who are contemplating the use of metformin. In reading the article by Faure et al., one could conclude that the use of metformin is detrimental to the future fecundity of the male fetus, in general. Our position is that this statement is limited only to supra-physiological doses of metformin - in pregnant mice, and that it is inappropriate to extrapolate the observation to humans in whom such high doses of metformin is never recommended in a clinical setting.

In general, and as reported in the Faure et al. references, metformin is usually used at doses of approximately 20 to 30 mg/kg in humans for the treatment of diabetes or polycystic ovary syndrome (PCOS), both conditions being associated with female infertility and several pregnancy complications. Pregnant women affected by obesity, diabetes and/or PCOS have an increased risk for the hypertensive disorders of pregnancy, including preeclampsia, and metformin can reduce such risk (3, 4). A recent systematic review of human studies demonstrates that metformin mediates several molecular pathways implicated in the pathogenesis of pre-eclampsia and intrauterine growth retardation (5). There is therefore a growing interest for the use of metformin in those high-risk women. Since preeclampsia and other placenta-mediated complications of pregnancy are often associated with fetal and maternal complications, including impaired development of male fetus genitalia, that can have a long-term deleterious impact on both mother and child, there is an urgent need to establish the safety of metformin in the fetus (6). In fact, a recent review of the literature suggests a lack of teratogenicity of metformin in pregnancy and a significant decrease in adverse effects including miscarriages and impaired embryo implantation with its use (7). The author also demonstrated that metformin could have positive effect on steroidogenesis and spermatogenesis in men with metabolic disorders and it should be considered for the improvement of male reproductive functions and fertility.

The study by Faure et al. used a metformin dose equivalent to 250 mg/kg/day in mice, a dose approximately 10 times higher than that typically used in humans, to study the impact of metformin on the gonadal function of the murine fetus exposed to metformin during pregnancy. We believe this observation should have then been followed by a second validation study including an evaluation of the dose-response effect using doses of metformin close to those usually used in humans. It is not uncommon for a beneficial exposure to become deleterious when the exposure is excessive. Faure et al. should have specified that excessive exposure to metformin during pregnancy in mice could potentially affect gonadal development in the male fetus, as opposed to generalizing a potential negative effect of metformin during pregnancy.

In conclusion, it is our hope that this study and our commentary will encourage additional analyses of existing studies, including a follow-up of children exposed to metformin during pregnancy. In addition, it would certainly be useful to consider studies in other animal models including dose-response assessments.

## Author Contributions

DC and EB conceived the commentary structure, performed the review of literature and finally revised the manuscript. All authors contributed to the article and approved the submitted version.

## Funding

The publication fees were funded by the Jeanne et J-Louis Levesque Perinatal Research Chair at Université Laval, Quebec, Canada.

## Conflict of Interest

The authors declare that the research was conducted in the absence of any commercial or financial relationships that could be construed as a potential conflict of interest.

## Publisher’s Note

All claims expressed in this article are solely those of the authors and do not necessarily represent those of their affiliated organizations, or those of the publisher, the editors and the reviewers. Any product that may be evaluated in this article, or claim that may be made by its manufacturer, is not guaranteed or endorsed by the publisher.
